# Automatic localisation and per-region quantification of traumatic brain injury on head CT using atlas mapping

**DOI:** 10.1016/j.ejro.2023.100491

**Published:** 2023-05-29

**Authors:** Carolina Piçarra, Stefan Winzeck, Miguel Monteiro, Francois Mathieu, Virginia F.J. Newcombe, Prof David K. Menon, Prof Ben Glocker

**Affiliations:** a Biomedical Image Analysis Group, Department of Computing, Imperial College London, London, UK; b Division of Neurosurgery, Department of Surgery, University of Toronto, Toronto, Ontario, Canada; c Interdepartmental Division of Critical Care Medicine, University of Toronto, Toronto, Ontario, Canada; d Division of Anaesthesia, Department of Medicine, University of Cambridge, Cambridge, UK

**Keywords:** Traumatic brain injury, CT, Image registration

## Abstract

**Rationale and objectives:**

To develop a method for automatic localisation of brain lesions on head CT, suitable for both population-level analysis and lesion management in a clinical setting.

**Materials and methods:**

Lesions were located by mapping a bespoke CT brain atlas to the patient’s head CT in which lesions had been previously segmented. The atlas mapping was achieved through robust intensity-based registration enabling the calculation of per-region lesion volumes. Quality control (QC) metrics were derived for automatic detection of failure cases. The CT brain template was built using 182 non-lesioned CT scans and an iterative template construction strategy. Individual brain regions in the CT template were defined via non-linear registration of an existing MRI-based brain atlas.

Evaluation was performed on a multi-centre traumatic brain injury dataset (TBI) (n = 839 scans), including visual inspection by a trained expert. Two population-level analyses are presented as proof-of-concept: a spatial assessment of lesion prevalence, and an exploration of the distribution of lesion volume per brain region, stratified by clinical outcome.

**Results:**

95.7% of the lesion localisation results were rated by a trained expert as suitable for approximate anatomical correspondence between lesions and brain regions, and 72.5% for more quantitatively accurate estimates of regional lesion load. The classification performance of the automatic QC showed an AUC of 0.84 when compared to binarised visual inspection scores. The localisation method has been integrated into the publicly available Brain Lesion Analysis and Segmentation Tool for CT (BLAST-CT).

**Conclusion:**

Automatic lesion localisation with reliable QC metrics is feasible and can be used for patient-level quantitative analysis of TBI, as well as for large-scale population analysis due to its computational efficiency (<2 min/scan on GPU).

## 1 Introduction

TBI is one of the leading causes of disability and death globally, with CT remaining the gold standard imaging modality for its initial assessment and treatment guidance [1], [2].

While space occupying characteristics of lesions are recognised to be of prognostic significance on several scoring systems [3], current approaches at volumetric lesion measurements such as ABC/2 score [4] are often inaccurate for traumatic hematomas, challenging to perform in the clinical setting and extremely time-consuming when looking at large-scale datasets [5]. Accurate calculation of injury burden via automatic volumetric measurements and location may help provide valuable information for patient management, and research, enabling for example stratification of patients in clinical trials [6].

Several computational methods have been developed to automatically quantify and characterise different lesion types, in order to develop a more reliable and time-efficient pipeline for the assessment of acute TBI CT scans and to inform prognosis models [7], [8].

However, the clinical utility of only providing overall lesion volume information is limited, as the spatial distribution of lesions has been shown to be relevant in the context of head lesion disorders [9], and significantly correlated to functional outcome [10], [11], [12], [13]. Isokuortti et al. [14] examined the distribution of subdural haematomas, subarachnoid haemorrhages and contusions in a representative sample (n = 3023) of CT scans from TBI patients. This analysis was non-automated and only classified a lesion as frontal, parietal, temporal, or occipital. To the best of our knowledge, most of past studies have either addressed TBI lesion location qualitatively [10], [15], [16], in a non-automated fashion [17], or were focused on other conditions [11], [13], [18]. For example, Ernst and colleagues [13] used atlas registration to calculate the overlap of ischemic stroke lesion volume with each brain region.

In this study we propose a processing pipeline for automated localisation and spatial association of brain lesions in CT. In contrast with previous studies, the output of our tool includes the volume of lesion affecting each brain region, which can be calculated in subject-specific space, for clinical assessment of each patient, or in atlas space, for population-level analysis. A segmentation map, the total lesion and brain volume, and the lesioned occupied volume in 31 brain regions, are computed in less than two minutes per scan. The results were evaluated by visual inspection of the atlas mapping by a trained expert. We also propose quality control (QC) metrics that support the curation and analysis of large datasets and automatic detection of failure cases.

The proposed lesion localisation approach was implemented as an extension of the Brain Lesion Analysis and Segmentation Tool for Computed Tomography (BLAST-CT, https://github.com/biomedia-mira/blast-ct/tree/master) [7], a deep learning-based method using convolutional neural networks for multiclass, voxel-wise segmentation and volumetric quantification of TBI lesions in CT. The original segmentation method classifies individual voxels as normal or abnormal tissue types. The proposed localisation component adds clinically important information about the location of pathology. To demonstrate the utility of this new tool for population-level analysis, a proof-of-concept spatial assessment of lesion prevalence was conducted, as well as an exploration of the distribution of lesion volume per brain region, stratified by clinical outcome.

## 2 Materials and methods

### 2.1 Datasets and procedures

The CT data used for this study were collected as part of the Collaborative European Neuro Trauma Effectiveness Research in TBI study (CENTER-TBI, NCT02210221) [19]. Patients were recruited at 65 different centres, in 18 countries, between Dec 9, 2014, and Dec 17, 2017. Acquisition parameters were not standardised across sites. The present work makes secondary use of fully anonymised data, and no additional ethics approval was required. The work was compliant with the Health Insurance Portability and Accountability Act.

The 1028 CT scans were divided into two datasets. Dataset 1 consists of 189 patients without abnormal findings and was used in the development of the lesion localisation method. Dataset 2 comprises 839 scans, acquired from 512 TBI patients with abnormal findings, and was used for validation, to evaluate the automatic QC of the pipeline and as part of the proof-of-concept population-level analyses.

The processes used to obtain the lesion segmentations of the scans of Dataset 2 were described in detail in previous work [7]. Each lesion segmentation map included four classes: intraparenchymal haemorrhages (IPH), which also includes small petechial haemorrhages; extra-axial haemorrhages (EAH): subdural haematomas, extradural haematomas, and traumatic subarachnoid haemorrhages; perilesional oedema; and intraventricular haemorrhages (IVH).

### 2.2 CT template construction for lesion localisation

Our lesion localisation approach makes use of a CT brain atlas which was specifically constructed as part of this work. Brain atlases have been used in previous registration-based pipelines for medical image segmentation. To reduce potential sources of registration error, an atlas should be as representative as possible of the images to be segmented [20]. While neuro-anatomical atlases are widely used in MRI-based studies [21], similar atlases for head CT are not readily available. Robustly registering pathological CT scans directly to an existing MRI atlas remains an open problem. It was thus important to remove the image modality gap and construct a bespoke CT brain atlas.

Using the scans from Dataset 1, we employ an iterative template construction strategy [22]. Seven scans were excluded as processing consistently failed due to corrupt image headers. We performed seven iterations, four with affine registration and three with deformable registration. In each iteration, the 182 CT scans were registered to an intermediate template image obtained at the end of the previous iteration by averaging over all registered scans. This iterative process results in an increasingly sharp template image, which is used as a target in the next iteration. In the very first iteration, when there is no CT-based template available, we use an MRI-based T1-weighted MNI template [23] as target. The result of this process is a study-specific CT template image, corresponding to the average of the 182 registered CT scans.

Subsequently, individual anatomical brain regions were defined in the CT template via a non-linear registration with the MRI-based MNI template, allowing the transfer of the fine-grained anatomical parcellations from an MNI-based atlas (construction process detailed in the appendix) to the CT template. The construction process of the parcellated MNI atlas is available in the appendix (p A). Due to the invertibility of the non-linear transformation, this also enables the transfer of information from the CT template space to the MNI space, which can be useful for population-level analysis. The registration parameters applied in all registration tasks described in this section are available in the appendix (p A).

### 2.3 Lesion localisation in head CT scans

**Fig. 1:**
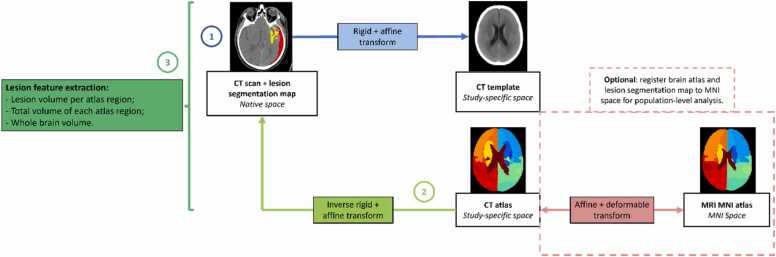
Flowchart of the full lesion localisation method. 1- Every native CT scan is registered to the CT template; 2- The inverse of the transformation calculated in step 1 is used to map the parcellated atlas to native patient space. 3- Relevant volumes are calculated from the overlap between the parcellated brain regions and each lesion segmentation map. Optionally, the brain atlas and the subject’s segmentation map can be registered to MNI space using the reversible non-linear transformation between our CT atlas and the MNI atlas, calculated during the CT template construction. This way the lesion volume values can be calculated from the overlap in a canonical neuroimaging space, which might be more suitable for population-level analysis.

Fig. 1 summarises the lesion localisation process: a patient CT scan is affinely registered to the CT template (1), and the inverse of this transformation is applied to map the anatomical brain parcellation back to the native patient space (2). Once in the same imaging space, the CT atlas brain regions and the lesion segmentation map can be overlayed and the volume of each lesion per brain region, the full volumes of the projected atlas regions and of the whole brain can be calculated (3). Additionally, the non-linear transformation between our CT atlas and the MNI atlas, which was established as part of the CT template construction, enables the mapping of the patient-specific lesion segmentation maps back to MNI space for population-level analysis in a canonical neuroimaging space.

The lesion localisation method was applied to all scans in Dataset 2, with four random seeds to increase the robustness of the initialisation of the registration algorithm, keeping the registration result that yields the highest image similarity between the registered patient scan and the CT template. This is measured as the correlation coefficient on image intensities. All final CT template registrations were visually inspected by a trained expert and rated with a score from 1 to 5. The scale was established based on the difficulty of aligning individual elements of the image. More specifically, the brain outline is the most straightforward component to align, followed by larger brain regions, and lastly, smaller and more variable regions such as ventricles. Accordingly, the scoring system was formulated as follows: a score of 1 indicating complete misalignment of the atlas; a score of 2, the misalignment of the brain outline; a score of 3, the alignment of the brain outline and most areas, while some significant areas, for instance, the ventricles or brainstem, remained misaligned; a score of 4 indicating satisfactory alignment of all regions, with minor misalignments; and a score of 5 implying excellent alignment. Results rated with a score of 3 or more were considered acceptable, as the general goal of this tool is to provide an approximate anatomical correspondence between lesions and brain regions, effectively and quantitatively, in order to enable further analysis. In studies that require accurate association of lesions and ventricles, a cut-off of 4 should be considered.

### 2.4 Quality control mechanisms

For each registration of a patient scan, we calculate an intensity-based similarity metric (SM) which quantifies how well the images visually correspond and thus provides a proxy of registration quality without requiring any annotations. This offers an effective way for automatic quality control (QC) of the localisation performance. An SM threshold was empirically established, below which the result was considered to be sub-optimal. As an additional test of the SM ability to discriminate between good and bad results, the area under the curve (AUC) was calculated when comparing the SM values with the binarised visual quality scores (not acceptable: 1–2, acceptable: 3–5).

Two additional empirical quality control rules were defined to facilitate the visual inspection of results and the flagging of potential failure cases. The outliers of the distribution of each brain region volume were identified, flagging scans that presented more than five outlier regions. Any scan with a volume of IVH higher than 1 mL localised outside the ventricles was also flagged as sub-optimal.

### 2.5 Application use-case: Spatial analysis of lesion prevalence and lesion volume stratified by outcome

To demonstrate the clinical utility of our lesion localisation component, the reference segmentations of Dataset 2 were used to create a prevalence map per lesion class, indicating how many subjects had a lesion volume higher than a defined threshold on each brain region. The minimum threshold applied should be 0.1 mL to exclude minor misalignments, as our methodology does not allow for voxel-level localisation accuracy. Furthermore, the distribution of the calculated lesion volumes per region was illustrated, stratifying by good (Extended Glasgow Outcome Scale (GOSE) > 7) and poor (GOSE < 7) patient outcome.

## 3 Results

### 3.1 Datasets

Upon the registration of all scans of Dataset 2 to the CT template, 7 scans were found to be corrupted and hence excluded. Table 1 shows the descriptive statistics for both datasets used.

**Table 1:** Cohort demographic and clinical information for both datasets used. Some percentages do not add up to 100 because of rounding. Count and percentages are presented for categorical variables while the median and corresponding range are used to describe continuous variables.

	Dataset 1 (n = 189)	Dataset 2 (n = 512)
**Age(years)**	55 (6–89)	58 (6–89)
**Biological sex**		
Female	101 (53%)	163 (32%)
Male	88 (47%)	349 (68%)
**Mechanism of injury**	---	
Acceleration or deceleration		111 (22%)
Blow to head or hit object		77 (15%)
Fall from height		208 (41%)
Multi-mechanistic		99 (19%)
Unknown		17 (3%)
**Glasgow Coma Scale**	---	
13–15 (Mild TBI)		299 (58%)
9–12 (Moderate TBI)		57 (11%)
9 (Severe TBI)		136 (27%)
Missing values		20 (4%)
**Time from injury to first CT scan (h)**	---	2.0 (0.2–77.0)
**Repeat scan done**	---	412 (80%)
**Time from injury to second CT scan (h)**	---	19 (0.9–190.0)
**Interval between CT scans (h)**	---	16.0 (0.1–190.0)
**Marshall Score**	---	
I		120 (23%)
II		234 (46%)
III		29 (6%)
IV		6 (1%)
V		2 (<1%)
VI		121 (24%)
**Lesion volume (mL)**	---	3.84 (0.00–208.00)
**Presence of:**	---	
Epidural haematoma		54 (11%)
Acute subdural haematoma		223 (44%)
Traumatic subarachnoid haemorrhage		313 (61%)
Intraventricular haemorrhage		88 (17%)
Intraparenchymal haemorrhage		224 (44%)
Cisternal compression		99 (19%)
Midline shift > 5 mm		71 (14%)
**Extended Glasgow Outcome Scale at 6 months**	---	
8 (Upper good recovery)		121 (24%)
7 (Lower good recovery)		78 (15%)
6 (Upper moderate disability)		67 (13%)
5 (Lower moderate disability)		59 (12%)
4 (Upper severe disability)		25 (5%)
3 (Lower severe disability)		59 (12%)
2 (Vegetative state)		0 (0%)
1 (Death)		66 (13%)
Missing values		37 (7%)

### 3.2 CT template construction for lesion localisation

**Fig. 2:**
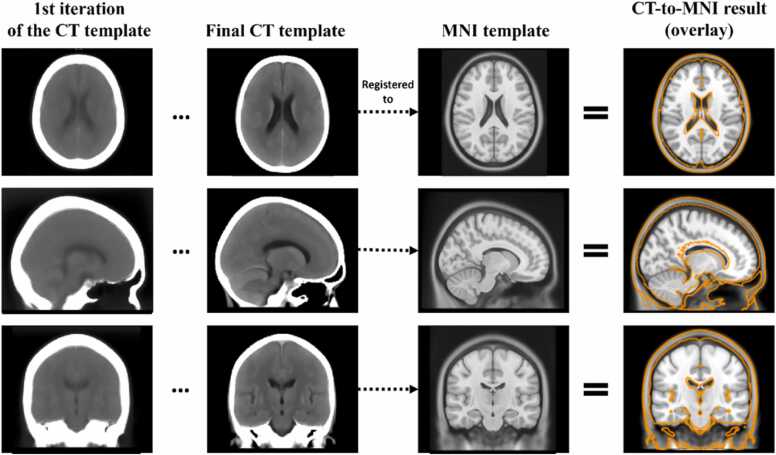
Initial and final CT template, i.e. resulting from the 1st and 7th iteration of the template construction process. The final template is then non-linearly registered to the MNI MRI template. The result of this CT-to-MNI registration is shown as the orange contour, overlaid on the MNI template image for verification of anatomical correspondence. This transformation was then used to map the brain regions, originally parcellated in MNI space, to the CT template space.

The first two columns of Fig. 2 show the evolution between the first and last iterations of the study-specific CT template construction, the latter having well-defined and visible anatomical structures. This final template was then registered to the MNI template (third column) and the result of the registration can be observed in the last column of Fig. 2, overlayed on the registration target (the MNI template). During the parameter optimisation process, a trade-off was found between achieving a satisfactory alignment of soft tissue or of the skull. The result shows a good ventricle alignment without significant skull deformation. Mitigating the slight discrepancy in overall brain size was deemed not a priority as the parcellated atlas regions were nonetheless dilated beyond the skull to ensure full coverage of the atlas across patient brains of different sizes, given that the CT atlas mapping to new patient scans is based on affine transformations.

### 3.3 Alignment of the parcellated atlas to native scans

**Fig. 3:**
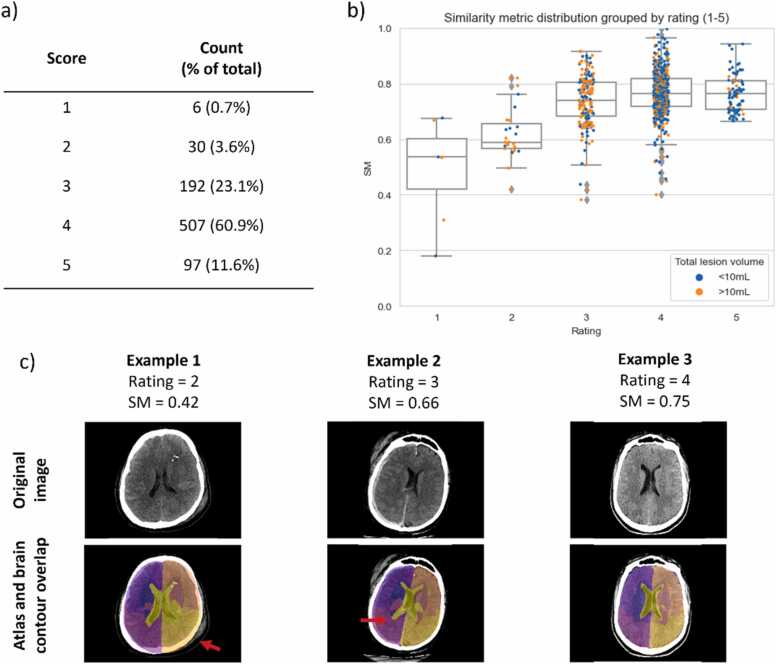
a) Count and percentage of results rated from 1 to 6. 1: Atlas completely misaligned; 2: Brain outline misaligned; 3: Brain outline and most regions aligned but relevant regions misaligned, e.g., ventricles or brainstem; 4: Acceptable alignment of all regions; 5: Good alignment; 6: Perfect alignment. b) Distribution of SM grouped by rating attributed by a trained expert. Each point on the strip plots per rating value represents a scan with a true total lesion volume over (orange) or under (blue) 10 mL; c) Atlas alignment of three examples, rated with 2, 3 and 4. Red arrows indicate the atlas misalignment features characteristic of each rating score.

**Fig. 4:**
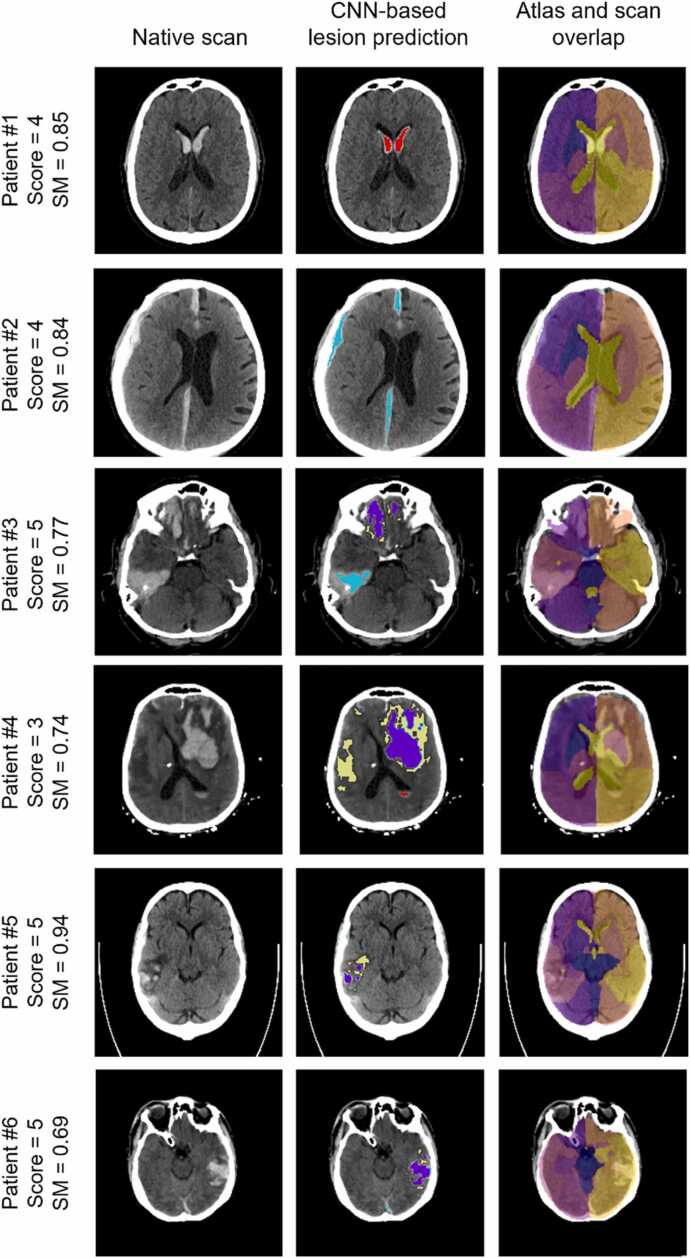
Qualitative atlas mapping results from Dataset 2, with corresponding manual score and SM value. Images in neurological orientation. Lesion map prediction (from BLAST-CT) colour legend: Red - IVH; Purple - IPH; Yellow - Oedema; Light blue – EAH.

The table in Fig. 3a) shows the percentage of scans manually rated from 1 to 5. The sub-figure b) includes SM boxplots grouped by each rating value. The strip plots per rating value aim to show the distribution of scans with reasonably large lesions (over 10 mL) for each rating value and over the SM range. The AUC when using the SM to separate samples in terms of acceptable results was found to be 0.84. Finally, sub-figure c) shows one example classified with each intermediate rating score, providing a better understanding of the visual criteria used when rating the results. Fig. 4 shows six results of the atlas mapping from Dataset 2. As it is based on affine registration, our tool is not always able to compensate for the level of distortion caused by some severe lesions, as it is the case of Patient 4, whose top right ventricle was collapsed by the lesion. However, it is capable of dealing with anatomical asymmetries (e.g., the ventricles of Patient 2).

### 3.4 Quality control mechanisms

The SM threshold was set to 0.65, which led to the identification of 96 sub-optimal results. Additionally, 12 scans were flagged due to having regions with outlier volumes and 16 scans had more than 1 mL of IVH outside of the ventricles.

### 3.5 Application use-case: Spatial analysis of lesion prevalence and lesion volume stratified by outcome

**Fig. 5:**
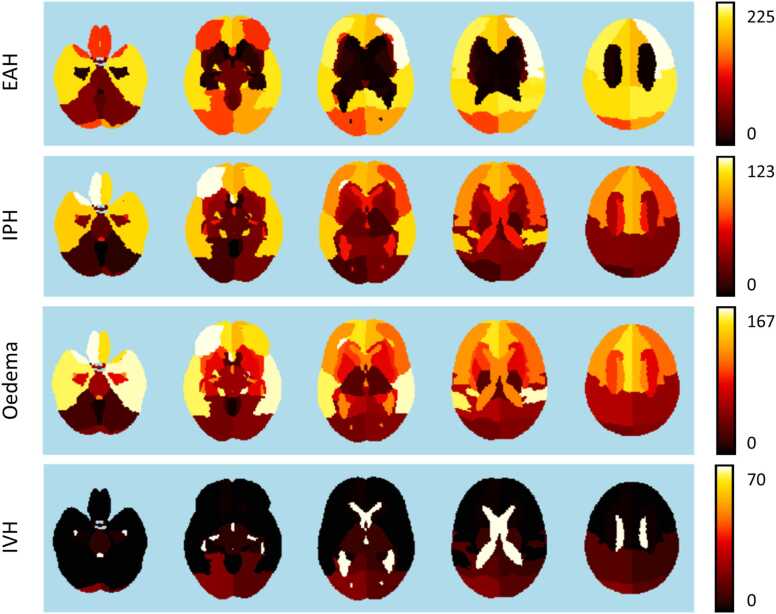
Per-class prevalence maps. All maps are displayed in neurological orientation. Threshold = 0.1 mL. The prevalence of EAH, IPH and oedema lesions is significantly higher in the anterior half of the brain, while IVH lesions are most prevalent in the ventricles. EAH also presents, as expected, higher prevalence in regions contiguous with the cerebral border.

Fig. 5 shows the prevalence maps for every lesion class, all thresholded at 0.1 mL. The prevalence values per brain region for both thresholds 0.1 mL and 1 mL are included in the Appendix (Table B.1.1). Only 3 subjects had a volume of IVH higher than 1 mL localised in regions outside the ventricles. This indicates that although 68 patients have a significant volume (>0.1 mL) of IVH in the surrounding regions of the ventricles, for 65 patients this volume ranges between 0.1 mL and 1 mL.

**Fig. 6:**
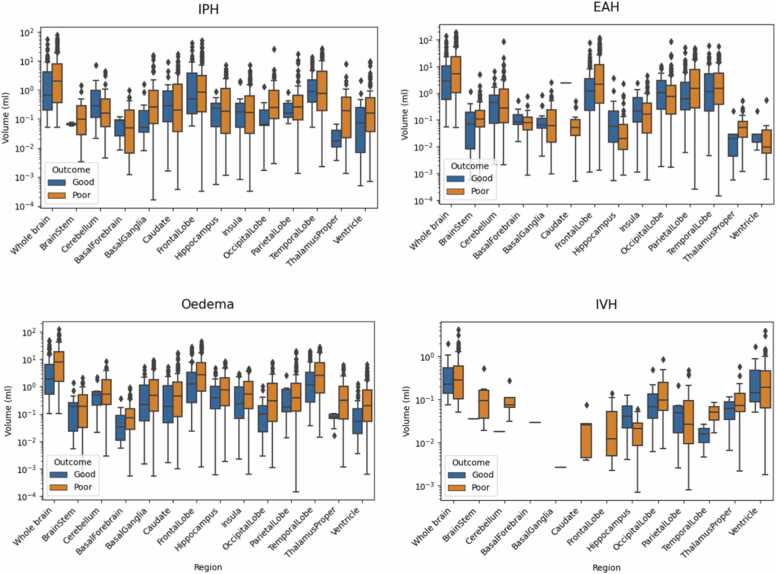
Per-class boxplots of lesion volume localised in the whole brain and each brain region, stratified by outcome. Volumes calculated from reference segmentation maps of Dataset 2 scans, excluding sub-optimal cases.

The distribution of the volumes calculated within each brain region, for all lesion classes and stratified by outcome, is shown in Fig. 6. Clear differences can be seen in the total lesion volume, as shown in the first boxplot of each subplot. However, such aggregation might miss relevant differences in individual regions that may be clinically relevant. Our tool allows for a more detailed assessment of which regions these differences come from.

## 4 Discussion

In this study, we proposed a registration-based approach for localisation of brain lesions on head CT scans using a bespoke CT template. The approach was tested on a large multicentre dataset, with a wide range of ages and a majority of mild TBI cases, representing the clinical reality of TBI prevalence [24].

We found that this tool could be used for patient-level spatial volumetric analysis of head lesions, with 95.7% of results rated by a trained expert as suitable for approximate anatomical correspondence between lesions and brain regions (rating ≥ 3), and 72.5% to obtain more quantitatively accurate estimates of regional lesion load (rating ≥ 4).

The establishment of several QC metrics, along with the time efficiency that affine registration allows, make this a suitable tool for population-level analysis in large-scale research studies. Besides the two potential population-level use cases presented, our tool could also be used to perform an appraisal of lesion segmentation tools and identify potential biases, by analysing the spatial distribution of its errors.

The AUC when using the SM to discriminate between good and poor results (i.e., results rated with higher and lower scores) was found to be 0.84. However, when only considering scans with accurate alignment of all regions as acceptable (rated 4 or 5), this AUC decreases to 0.61. This demonstrates that although the SM might be a considerably effective way to classify the overall quality of the atlas mapping results, it is not precise enough to translate errors in smaller regions such as the ventricles, likely due to the lack of physical significance of this metric. Therefore, it might be important to pair this metric with the third one mentioned above, i.e., excluding scans with a volume of IVH located outside of the ventricles, or even with a significant volume of IPH, EAH or oedema inside the ventricles, both situations which would be anatomically implausible.

A limitation of this study is its reliance on a sole trained expert to manually assess the findings. To evaluate observer variability, further validation is required in future work, particularly given the unavailability of other quantitative evaluation metrics (e.g. dice scores) due to the lack of reference brain region segmentations.

The spatial distribution of IPH, as well as the presence of underlying EAH and oedema, have been shown to be correlated with outcome and lesion progression [10], [25]. However, most studies on this topic have solely used either a dichotomous variable to indicate presence or the volume in each brain lobe. The finer grain localisation enabled by our tool, as well as the possibility to use any anatomical atlas, may provide important new insights that were previously unavailable at a population level.

It was shown that, although this pipeline is subject to the limitations of affine registration, not allowing for voxel-level precision and being subject to registration errors that are challenging to measure precisely, it can localise lesions accurately even in considerably deformed scans. It is therefore suitable for further guidance of patient-specific volumetric assessment of TBI lesions in clinical settings, facilitating diagnosis, treatment, and prognosis decisions. Future work might focus on the integration of a partial deformable registration, for regions of challenging alignment, as well as on the creation of a patient-specific report that translates the outcomes of our algorithm in a clinically meaningful way. Our pipeline is integrated into a publicly available lesion segmentation tool (https://github.com/biomedia-mira/blast-ct/tree/master). We hope this will encourage researchers to use the method more extensively and thereby provide further evidence of its clinical utility.

## Funding statement

This project received funding from the European Research Council (10.13039/100010663ERC) under the European Union’s Horizon 2020 research and innovation programme (Grant Agreement No. 757173, Project MIRA). The CENTER-TBI study was supported by the 10.13039/100011102European Union 7th Framework Programme (EC grant 602150). CP and SW were supported by the UKRI London Medical Imaging & Artificial Intelligence Centre for Value Based Healthcare. VFJN was supported by an 10.13039/501100000691Academy of Medical Sciences/The Health Foundation Clinician Scientist Fellowship.

## Ethical statement

The authors declare no competing interests. The data used in this study were from the Collaborative European Neuro Trauma Effectiveness Research in TBI study (CENTER-TBI). Ethical approval was obtained in accordance with all relevant local regulations for each recruiting site, and informed consent by patients, or their legal representative/relatives was obtained according to local laws and regulations. Clinical data was accessed via the Neurobot platform (RRID/SCR_017004, core data, version 3.0; International Neuroinformatics Coordinating Facility; released November 24, 2020). A complete ethics statement, which contains a comprehensive list of sites, ethical committees, and approval numbers, is available online at https://www.center-tbi.eu/project/ethical-approval.

## CRediT authorship contribution statement

**Monteiro Miguel:** Conceptualization, Data curation, Software, Supervision. **Mathieu Francois:** Data curation, Resources, Writing – review & editing. **Picarra Carolina:** Data curation; Formal analysis; Investigation; Methodology; Software; Validation; Visualization; Roles/Writing - original draft; Writing - review & editing. **Winzeck Stefan:** Conceptualization, Data curation, Methodology, Resources, Supervision. **Glocker Ben:** Conceptualization; Data curation; Formal analysis; Funding acquisition; Investigation; Methodology; Project administration; Resources; Supervision; Roles/Writing - original draft; Writing - review & editing. **Newcombe Virginia:** Data curation; Conceptualization; Supervision; Resources; Writing - review & editing. **Menon David:** Conceptualization; Project administration; Supervision; Writing - review & editing.

## Declaration of Competing Interest

The authors declare the following financial interests/personal relationships which may be considered as potential competing interests: Prof. Ben Glocker reports financial support was provided by European Research Council (ERC) under the European Union’s Horizon 2020 research and innovation program (Grant Agreement No. 757173, Project MIRA). The CENTER-TBI study was supported by the European Union 7th Framework Programme (EC grant 602150). Carolina Piçarra and Stefan Winzeck report financial support provided by UKRI London Medical Imaging & Artificial Intelligence Centre for Value Based Healthcare. Virginia Newcombe reports financial support was provided by Academy of Medical Sciences.
