# Supplementary material for: Automatic localisation and per-region quantification of traumatic brain injury on head CT using atlas mapping
Source: Eur J Radiol Open. 2023 May 29;10:100491. doi: 10.1016/j.ejro.2023.100491 (PMC10241839; doi:10.1016/j.ejro.2023.100491)
Supplement: Supplementary file 1 — Supplementary material [file mmc1.docx]

# Supplementary Material/Appendix:

1. **Methods**

## Implementation details

This lesion localisation methodology was fully implemented using the Python programming language with the following packages: os ([https://docs.python.org/3/library/os.html;](https://docs.python.org/3/library/os.html) last accessed: 1/08/2021), SimpleITK ([simpleitk.org;](http://simpleitk.org/) last accessed: 31/08/2021), numpy ([numpy.org](http://numpy.org/); last accessed: 31/08/2021), pandas ([pandas.pydata.org;](http://pandas.pydata.org/) last accessed: 31/08/2021) and operator ([https://docs.python.org/3/library/operator.html;](https://docs.python.org/3/library/operator.html) last accessed: 1/08/2021).

## A.2 Generation of the parcellated MNI atlas:

The parcellated MRI-based MNI atlas used in our study was previously constructed using 652 MR T1- weighted scans from the Cam-CAN study [26], parcellated via MALP-EM [27]. Patient-specific age- unbiased segmentations of cortical regions and ventricles were obtained through the projection of individual region atlases to the standard MNI MRI template. Distance maps were then used in order to map each WM voxel to its closest region, followed by the fusion of regions based on prior anatomical knowledge (e.g., medial frontal cortex was assigned to the medial frontal lobe). This resulted in a coarser subdivision of the brain into 31 different regions (Figure A.2.1). A list of all the parcellated regions is available in the left column of Table B.1.1.

| 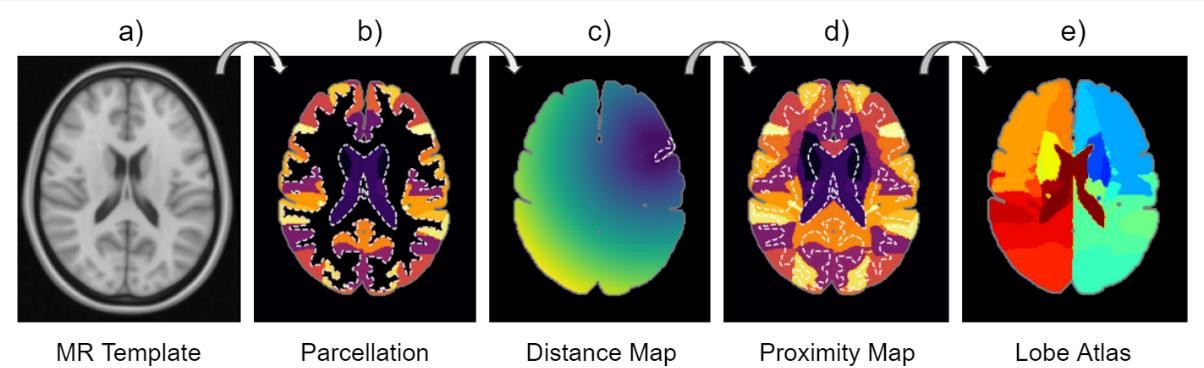 |
| --- |
| Figure A.2.1: Method for the construction of the parcellated MNI atlas. Segmentations of cortical regions and ventricles were obtained through the projection of patient-specific region atlases to the standard MNI MRI template (b)). Distance maps (c)) were then used to map each WM voxel to its closest region (d)), followed by the fusion of regions based on prior anatomical knowledge (e)). |

## Registration parameters of the CT template construction

| Table A.3.1. Parameters employed when applying the antsRegistration function for the affine and non-linear registration  of native CT scans to every target used during the CT template construction. SyN: symmetric image normalisation method |
| --- |
| 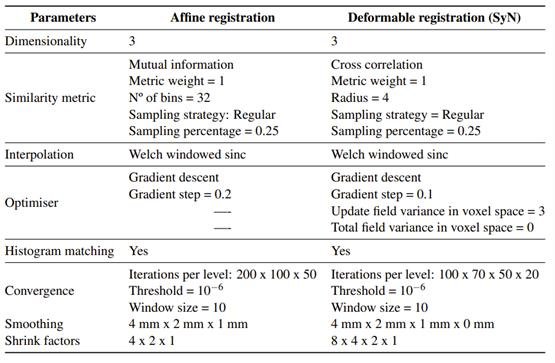 |

- 1. **Parameters of the registration of the CT template to MNI space**

Although the goal was to complete the full process in native python - using therefore only SimpleITK for the image registration - it was only possible to achieve a satisfactory alignment of both the ventricles and skull through the use of ANTs to calculate the deformable transformation.

To initialise the affine transformation, the centres of the two images were aligned. Mattes mutual information was set as the SM and the sampling strategy as regular, with a percentage of 25%. A gradient-based optimiser was selected (Gradient Descent Line Search) with a learning rate of 1, 100 iterations, a convergence minimum value of 10^-5^ and window size of 5. Missing values were interpolated linearly. A three-level multi-resolution pyramid was applied, setting the corresponding shrink factors and smoothing sigmas to [4, 2, 1] and [4, 2, 1] mm, respectively. Using this affine transformation as initialisation, a symmetric image normalisation method (SyN) transform was constructed with a gradient descent optimiser (learning rate= 0.1). The convolution kernel for both the update and total field variance are defined as 3 and 0 (specified in terms of the radius in voxel space), respectively. Cross correlation was set as the SM with a metric weight of 1 and a radius of 5. No sampling strategy was defined. A four-level multi-resolution registration approach was used, with 400, 200, 100 and 50

iterations for each level. The convergence threshold was set as 10−6 and window size as 5. The shrink factors and sigmas of gaussian smoothing for each level were defined as [6, 4, 2, 1] and [4, 2, 1, 0] mm, respectively.

## Parameters of the registration of native scans to the CT template

This registration was fully completed with SimpleITK. The rigid Euler 3D transform was initialised by aligning the two images’ geometric centres. Mattes mutual information was set as the SM and the sampling strategy as regular, with a percentage of 20%. A gradient based optimiser was selected

(Gradient Descent Line Search) with a learning rate of 0.1, 200 iterations, a convergence minimum value of 10^-6^ and window size of 5. Missing values were interpolated linearly. A three-level multi-resolution registration approach was used, setting the corresponding shrink factors and smoothing sigmas to [4, 2, 1] and [4, 2, 1] mm, respectively. The following affine registration was constructed using the same parameters, except for the initialisation (i.e., SM, sampling, optimiser, interpolator and multi-resolution level parameters). The previously found rigid transformation was set as the moving initial transform, while an empty affine transformation was set as the initial transform to be modified during the optimisation process.

# Results

## Application use-case: Spatial analysis of lesion prevalence

| Table B.1.1: Per-region prevalence values for each lesion type. Prevalence values are obtained by initialising  a counter for every atlas region. Going through all the subjects, the counter is increased by 1 if the subject has a volume of that lesion class on that region higher than the defined threshold. |
| --- |
| 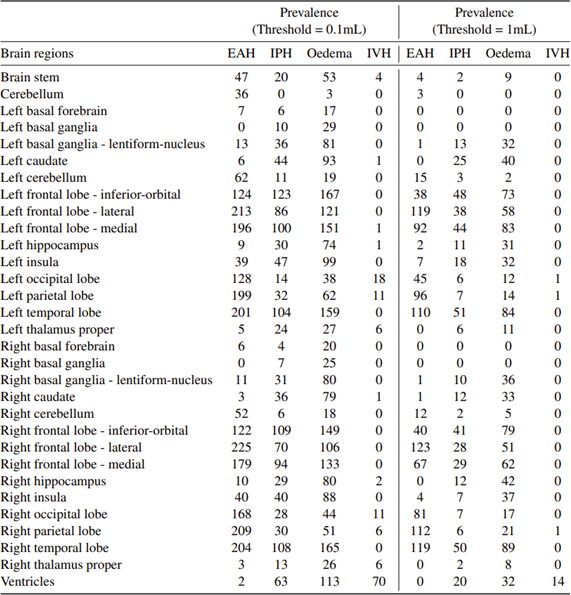 |
